# TNF-α promoter polymorphisms (G-238A and G-308A) are associated with susceptibility to Systemic Lupus Erythematosus (SLE) and *P*. *falciparum* malaria: a study in malaria endemic area

**DOI:** 10.1038/s41598-019-48182-5

**Published:** 2019-08-13

**Authors:** Harishankar Mahto, Rina Tripathy, Biswa Ranjan Meher, Birendra K. Prusty, Meenakshi Sharma, Divya Deogharia, Anjana Kumari Saha, Aditya K. Panda, Bidyut K. Das

**Affiliations:** 1 Department of Bioscience and Bioinformatics, Khallikote University, GMax Building, Konisi, Berhampur, 761008 Odisha India; 2 Centre for Life Sciences, Central University of Jharkhand, Brambe, Ranchi, 835205 Jharkhand India; 3 Department of Biochemistry, S.C.B. Medical College, Cuttack, 753007 Odisha India; 4 Computational Biology and Bioinformatics Laboratory, Department of Botany, Berhampur University, Berhampur, Odisha 760007 India; 5 Infectious Disease Biology Group, Institute of Life Sciences, Bhubaneswar, Odisha India; 6 Department of Medicine, S.C.B. Medical College, Cuttack, 753007 Odisha India

**Keywords:** Structural variation, Autoimmunity

## Abstract

Tumor necrosis factor-α (TNF-α) is a proinflammatory cytokine associated with autoimmune and infectious diseases. Importance of TNF-α in *P*. *falciparum* malaria and systemic lupus erythematosus (SLE) have been demonstrated. However, association of functional promoter variants with SLE and malaria is lacking in malaria endemic population. A total of 204 female SLE patients and 224 age and sex matched healthy controls were enrolled in the study. Three hundred fourteen *P*. *falciparum* infected patients with different clinical phenotypes were included. TNF-α polymorphisms (G-238A & G-308A) were genotyped by PCR-RFLP. Plasma levels of TNF-α was quantified by ELISA. Heterozygous mutants and minor alleles of TNF-α (G-238A and G-308A) polymorphisms were significantly higher in SLE patients compared to healthy controls and associated with development of lupus nephritis. In addition, both promoter variants were associated with severe *P*. *falciparum *malaria. SLE patients demonstrated higher levels of plasma TNF-α compared to healthy controls. TNF-α (G-238A and G-308A) variants were associated with higher plasma TNF-α. In conclusion, TNF-α (G-238A & G-308A) variants are associated with higher plasma TNF-α levels in SLE patients residing in malaria endemic areas and could be a contributing factor in the development of SLE and susceptibility to severe *P*. *falciparum* malaria.

## Introduction

Tumor necrosis factor-alpha (TNF-α) is a pro-inflammatory cytokine produced by wide range of cells such as macrophages, B cells, T cells and mast cells^1^. TNF-α is primarily produced as a trans-membrane protein that gets released from the membrane by a metalloprotease- TNF alpha converting enzyme (TACE), to form soluble 17 kDa protein^2^. TNF-α is a pleotropic cytokine with wide range of biological functions: it can initiate host defense against infectious diseases and along with it involved in toxicity and inflammatory processes^1^. TNF-α exerts its biological effect through specialized types of receptors viz. TNF receptor 1 (TNFR-1) and TNFR-2^3^. Expression of TNF receptors is tissue specific. TNFR1 is normally observed in most tissues but TNFR2 is restricted to cells of the immune system^3^. TNF-α has both a beneficial and deleterious role and it has been linked with infectious diseases and autoimmune disorders^4–7^. The TNF-α gene is located in short arm of chromosome 6 at position 21.3 and spans about 12 kilobase (kb) length^8^. Till date, 43 single nucleotide polymorphisms (SNPs) at promoter region of TNF-α gene with minor allele frequency data have been reported (https://www.ncbi.nlm.nih.gov/SNP/snp_ref.cgi?locusId=7124). Although there are contradictory reports, some SNPs at promoter region of *TNF-α* have been shown to regulate TNF-α expression and/or soluble TNF-α levels viz. TNF-α G-238A (rs361525), TNF-α G-308A (rs1800629), TNF-α T-857C (rs1799724), and TNF-α T-1031C (rs1799964)^9^. However, large number of genetic association studies have focused on two common promoter polymorphisms of TNF-α gene (G-238A and G-308A) and these have shown a significant association with SLE as well as *P*. *falciparum* infection in different populations^10,11^.

Malaria infection is believed to be an important selection pressure during human evolution and subjects with possible survival advantage genotypes against lethal malaria are more prevalent in malaria endemic areas^12^. This was true across the continents where malaria was endemic. *Plasmodium falciparum* infection is a life-threatening disease with diverse clinical manifestations^13–15^. TNF-α is an important molecule that works like a double-edged sword in malaria infection^16^. TNF-α can protect an individual against severe infection^17^ but when production is unregulated it could be damaging to the host. Low levels of TNF-α has been associated with susceptibility to *P*. *falciparum* infection. While various reports have demonstrated elevated plasma levels of TNF-α in severe malaria compared to uncomplicated infection^18,19^. Mortality in *P*. *falciparum* infection is also associated with very high plasma levels of TNF-α^18,20^. These observations collectively indicate the importance of TNF-α in *P*. *falciparum* malaria: optimum levels are essential for protection against infection. Recently, numerous studies have been carried out in different populations to established possible link between TNF-α gene polymorphisms and susceptibility/resistance to *P*. *falciparum* infection and/or clinical severity^21–24^. Most of the reports^22–24^ have included TNF-α promoter polymorphisms, that are believed to affect mRNA expression and alter plasma levels of protein molecule. TNF-α (G-308A) mutants have been associated with susceptibility to *P*. *falciparum* infection^23^, higher levels of parasitaemia^22^ and severe malaria^25^. However, an independent study on South-West Nigerian infected patients failed to demonstrate such association^24^. Another common TNF-α promoter (G-238A) variant is also linked to elevated parasitaemia^22^ and severe *P*. *falciparum* malaria^24^.

SLE is characterized by production of autoantibodies against self-antigens, formation of immune complexes, deposition of these complexes in tissues leading to organ damage and its failure^26^. Lupus nephritis remains one of the severe clinical manifestations and contributes to significant morbidity and mortality^27,28^. About 50–60% of SLE patients present with kidney dysfunction and the rate of renal affection is higher in Asian population^29^. Although type I interferons have been shown to play an important role in the pathogenesis of lupus nephritis^30^, there are cumulative evidence to suggest that TNF-α may also have a crucial role in renal dysfunction^6,31^. This has been demonstrated in the mouse model of SLE (MRL/lpr): elevated levels have been reported in serum and kidney tissue such as glomeruli, vascular smooth muscle, perivascular infiltrating cells and tubular epithelial cells^32,33^. Furthermore, the severity of proteinuria has been found to correlate with the degree of TNF-α expression in the kidney^32^. There are several reports of elevated TNF-α in SLE patients^34–37^. In a study involving African American, European American and Hispanic American SLE patients, high levels of plasma TNF-α was observed and a positive correlation with IFN-α was demonstrated^37^. A significant positive correlation between plasma TNF-α, with clinical severity and anti-ds DNA has also been reported in several studies^34–36^. Interestingly, TNF-α expression was found to be high in the renal tissue of patients with lupus nephritis^38,39^. Defective clearance of apoptotic bodies has been suggested to be an important factor in the pathogenesis in SLE^40,41^. TNF-α has been shown to induce apoptosis^42–44^. Elevated TNF-α in SLE patients could be one of the reasons for increased apoptosis, elevated production of nuclear debris followed by defective clearance of dead or dying cells. However, anti TNF-α therapy has provided no therapeutic advantage in the treatment of SLE^45,46^.

Importance of TNF-α in *P*. *falciparum* malaria has been widely investigated^16,47,48^ and it has been demonstrated that TNF-α is an important molecule for parasite clearance^49^. However, uncontrolled production of this cytokine during *P*. *falciparum* infection can lead to clinical complications^50,51^. In malaria endemic areas, subjects with moderate TNF-α producing genotypes have survival advantage^12^. The association of TNF-α in mouse models of lupus^6,52^ and in the clinical manifestations in humans has been documented^34–37^. For instance, higher expression of TNF-α has been associated with lupus nephritis^38,39^. Since TNF-α appears to be important to some aspects of the pathogenesis in both SLE and *P*. *falciparum* malaria, we hypothesized a possible relationship between TNF-α promoter variants with predisposition to SLE, notably lupus nephritis, in patients residing in a malaria endemic area.

There are limited studies in the Indian population^10,53,54^, especially, in the malaria endemic belts, for possible association between TNF-α polymorphisms (G-238A and G-308A) and SLE. A recent study has shown a significant association between heterozygotes and minor allele to SLE^10^. A study from south of India demonstrated an association between TNF-α promoter haplotype and protection against SLE^53^. In our study, we have enrolled SLE patients and controls from malaria endemic areas of Eastern India and investigated the association of TNF-α promoter variants with SLE. Furthermore, we have quantified plasma levels of TNF-α to assess the genotype-phenotype relationship. The novelty of our study relies on the enrolment of SLE patients and *P*. *falciparum* infected cases from malarial endemic areas. The question we have addressed is whether individuals from malarial endemic areas are vulnerable to the development of SLE if genetically susceptible.

## Results

### Baseline characteristics

In the present study, we enrolled 428 female subjects (224 healthy controls and 204 SLE patients) and 314 *P*. *falciparum* infected patients including 103 uncomplicated malaria (UM), 68 cerebral malaria (CM), eighty four multi organ dysfunctions (MODs) and 59 non-cerebral severe malaria (NCSM) (Table 1).

**Table 1:** Clinical baseline characteristics of SLE patients, *P*. *falciparum* infected cases and healthy controls.

Clinical profiles	SLE (n = 204)	Healthy control (n = 224)	*P*. *falciparum* infected patients (n = 314)			
			UM (n = 103)	CM (n = 68)	MOD (n = 84)	NCSM (n = 59)
Sex (male/female)	0/204	0/224	84/19	52/16	69/15	47/12
Age in years (mean ± SD)	27.84 ± 8.83	29.56 ± 5.48	33.18 ± 13.60	32.91 ± 14.89	34.54 ± 14.24	33.36 ± 13.28
Duration of disease in years (mean ± SD)	2.07 ± 1.13	—	—	—	—	—
*ACR criteria*						
Photosensitivity rash	63 (31)	—	—	—	—	—
Malar rash	84(41)	—	—	—	—	—
Discoid rash	28 (14)	—	—	—	—	—
Oral ulcer	97 (48)	—	—	—	—	—
Arthritis	103 (50)	—	—	—	—	—
NPSLE	12 (6)	—	—	—	—	—
AIHA	6 (3)	—	—	—	—	—
Serositis	9 (4)	—	—	—	—	—
Nephritis	83 (41)	—	—	—	—	—
Pneumonitis	9 (4)	—	—	—	—	—

Note. Data are no. (%) of participants unless otherwise specified. NPSLE, Neuropsychiatric systemic lupus erythematosus; AIHA, autoimmune hemolytic anemia.

### Prevalence of TNF-α promoter (G-238A & G-308A) polymorphisms

Prevalence of heterozygous (GA) and homozygous mutants (AA) for G-238A polymorphism was 12% and 1% respectively (Table 2). Similarly, GA and AA genotype frequency for G-308A polymorphism was 11% and 2% respectively. Distributions of TNF-α(G-308A) polymorphism in healthy female controls deviated from Hardy-Weinberg equilibrium (HWE) (G-308A: χ^2^ = 11.35, P value = 0.0007; G-238A: χ^2^ = 3.5, P value = 0.061).

**Table 2:** Distribution of TNF-α (−308G/A and −238G/A) polymorphisms in SLE patients and healthy controls.

SNPs	Genotype or allele	HC (n = 224)	SLE (n = 204)	P value	OR (95% CI)
G-238A	GG	195 (87)	159 (78)	1	Ref.
	GA	26 (12)	43 (21)	0.008	2.02 (1.19–3.44)
	AA	3 (1)	2 (1)	1.000	0.81 (0.13–4.95)
	G	416 (93)	361 (88)	1	Ref.
	A	32 (7)	47 (12)	0.032	1.69 (1.05–2.71)
G-308A	GG	194(87)	153 (75)	1	Ref.
	GA	25 (11)	43 (21)	0.005	2.18 (1.27–3.73)
	AA	5 (2)	8 (4)	0.261	2.02 (0.65–6.32)
	G	413 (92)	349 (86)	1	Ref
	A	35 (8)	59 (14)	0.002	1.99 (1.28–3.10)

Note: Data are no. (%) of participants unless otherwise specified. HC = healthy control; SLE = systemic lupus erythematosus; OR = odds ratio; 95% CI = 95% confidence interval.

### TNF-α (G-238A and G-308A) polymorphism are associated with SLE

As shown in Table 2, the prevalence of GA and minor allele ‘A’ for TNF-α (G-308A) polymorphism were significantly high in SLE patients compared to healthy controls (GA: P = 0.005, OR = 2.18; A: P = 0.002, OR = 1.99). Similarly, frequency of GA for TNF-α (G-238A) polymorphism were more frequent in SLE patients than healthy female controls (P = 0.008, OR = 2.02). Although minor allele for TNF-α (G-238A) polymorphism was more frequent in SLE patients compared to controls, the difference was not significant after Bonferroni correction (P = 0.032, OR = 1.69).

Furthermore, haplotype analysis (G-308A/G-238A) showed significantly higher prevalence of A-G and A-A in SLE patients compared to healthy controls (A-G: P = 0.049, OR = 1.63; A-A: P = 0.029, OR = 2.57) (Supplementary Table 1).

### Distribution of TNF-α (G-238A and G-308A) polymorphism in patients with nephritis

Since the study revealed a significant association between TNF-α promoter (G-238A and G-308A) polymorphisms and SLE, we analyzed the association of these polymorphisms with organ involvement. Lupus nephritis was the most important clinical phenotype observed and often associated with increased mortality in SLE. In our study, 41% of SLE patients had lupus nephritis. We categorized the patients into two broad groups: (1) Patients with lupus nephritis (LN+), and (2) patients without nephritis (LN−). As depicted in Table 3, GA genotype and minor allele (A) of TNF-α (G-238A) polymorphism was more frequent in patients with lupus nephritis (LN^+^) compared to those patients without renal involvement (LN^−^) (GA: P = 0.002, OR = 2.89;A: P < 0.001, OR = 2.92). However, distribution of TNF-α (G-308A) polymorphism was comparable among both groups.

**Table 3:** Prevalence of TNF-α (G-308A and G-238A) polymorphisms in lupus nephritis and non-nephritis SLE patients.

SNPs	Genotype or allele	LN^−^ (n = 121)	LN^+^ (n = 83)	P value	OR (95% CI)
G-238A	GG	104 (86)	55 (66)	1	Ref.
	GA	17 (14)	26 (31)	0.002	2.89 (1.44–5.78)
	AA	0 (0)	2 (3)	0.123	9.41(0.44–199.7)
	G	225 (93)	136 (82)	1	Ref.
	A	17 (7)	30 (18)	<0.001	2.92 (1.55–5.49)
G-308A	GG	97 (80)	56 (66)	1	Ref.
	GA	19 (16)	24 (30)	0.034	2.18 (1.10–4.34)
	AA	5 (4)	3 (4)	1.000	1.03 (0.23–4.51)
	G	213 (88)	136 (82)	1	Ref
	A	29 (12)	30 (18)	0.11	1.62 (0.93–2.82)

Note: Data are no. (%) of participants unless otherwise specified. LN^+^  = lupus nephritis; LN^−^ = non-lupus nephritis; OR = odds ratio; 95% CI = 95% confidence interval.

### Plasma TNF-α level in SLE patient & healthy controls

**Figure 1:**
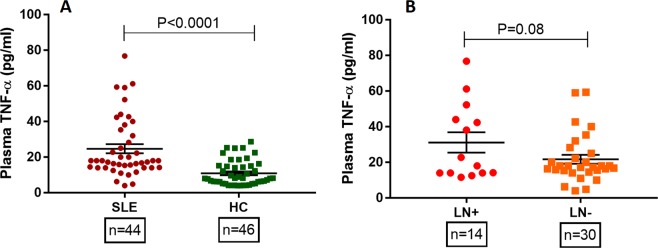
Plasma TNF-α levels in SLE patients and healthy controls. (**A**) Plasma TNF-α levels was quantified by ELISA in SLE patients (n = 44) and healthy controls (n = 46) and the mean TNF-α were compared by student’s t test. SLE patients displayed significantly higher TNF-α levels compared to healthy controls (P < 0.001). (**B**) SLE patients were categorized in to two broad group, presence (n = 14) or absence of kidney involvement (n = 30) and mean TNF-α levels was compared. P value less than 0.05 was considered as significant. LN^+^: lupus nephritis patients; LN^−^: SLE patients without kidney involvement.

We quantified plasma TNF-α in 90 samples (SLE: 44, HC: 46) by ELISA and mean TNF-α in each group was compared by unpaired ‘t’ test. SLE patients had significantly higher levels of TNF-α compared to healthy controls (P < 0.0001). Plasma levels of TNF-α were compared between LN^+^ and LN^−^ by student’s t test and results are shown in Fig. 1. The difference in mean TNF-α levels among LN+ and LN^−^ SLE patients was not statistically significant (P = 0.08).

### Genotype-phenotype association of TNF-α (G-238A and G-308A) polymorphisms

**Figure 2:**
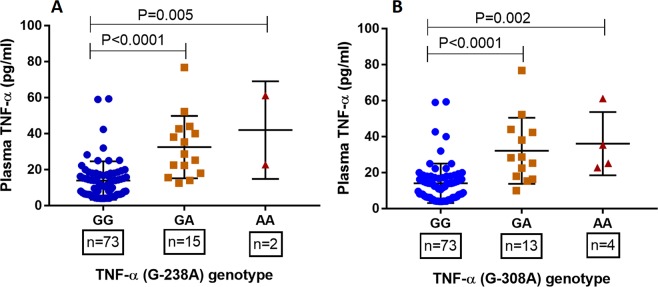
Association between TNF-α polymorphisms and levels of plasma TNF-α in SLE patients and control. Plasma TNF-α levels was measured by ELISA, based on availability of plasma samples (SLE: n = 44; HC: n = 46), and correlated with TNF-α genotypes (**A)** G-238A polymorphism and (**B**) G-308A polymorphism). Mean plasma TNF-α levels of various genotypes was compared by ANOVA followed by tukey’s multiple comparison post-test. P value less than 0.05 was considered as significant.

Several studies have demonstrated a functional relevance of TNF-α promoter polymorphisms (G-238A and G-308A) with expression of TNF -α. We compared plasma levels of TNF-α among different genotypes of TNF-α (G-238A and G-308A). As shown in Fig. 2A,B, for both promoter polymorphism (G-238A and G-308A) the major genotype GG expressed significantly lower levels of plasma TNF-α compared to heterozygous mutant (GA)(P < 0.0001) and homozygous minor genotypes (AA) (G-238A: P = 0.005; G-308A: P = 0.002). Furthermore, we analyzed association of both promoter polymorphism with plasma levels of TNF-α in SLE patients and healthy controls independently (data not shown) and interestingly the observations remained consistent.

### Association of TNF-α (G-238A and G-308A) polymorphisms with P. falciparum malaria

Association between TNF-α (G-238A and G-308A) polymorphisms and susceptibility to *P*. *falciparum* malaria have been well documented. In the present study, we enrolled 314 *P*. *falciparum* infected cases comprising of 103 uncomplicated cases and 211 severe malaria patients and genotyped for TNF-α (G-238A and G-308A) polymorphisms. As shown in Table 4, heterozygous genotype for TNF-α (G-238A) polymorphism and minor allele of G-308A polymorphism were more frequent in SM than UM (GA: P = 0.02, OR = 2.09; A: P = 0.02, OR = 2.05).

**Table 4:** Distribution of TNF-α (G-238A and G-308A) polymorphisms in *P*. *falciparum* malaria.

TNF-α polymorphisms		Clinical categories, (%) of subject				UM Vs CM		UM Vs MOD		UM Vs NCSM		UM Vs SM	
TNF-α G-238A	UM (n = 103)	CM (n = 68)	MOD (n = 84)	NCSM (n = 59)	SM (n = 211)	OR (95% CI)	P Value	OR (95% CI)	P Value	OR (95% CI)	P value	OR (95% CI)	P value
GG	88 (85)	50 (73)	56 (67)	50 (84)	156 (74)	1	Ref	1	Ref	1	Ref	1	Ref
GA	14 (14)	17 (25)	27 (32)	8 (14)	52 (25)	2.13 (0.97 to 4.70)	0.06	**3**.**03 (1**.**46 to 6**.**27)**	**0**.**002**	1.00 (0.39 to 2.56)	1.00	**2**.**09 (1**.**09 to 3**.**99)**	**0**.**02**
AA	1 (1)	1 (2)	1 (1)	1 (2)	3 (1)	1.76 (0.10 to 28.77)	1.00	1.57 (0.09 to 25.65)	1.00	1.76 (0.10 to 28.77)	1.00	1.69 (0.17 to 16.52)	1.00
G	190 (92)	117 (86)	139 (83)	108 (91)	367 (87)	1	Ref	1	Ref	1	Ref	1	Ref
A	16 (8)	19 (14)	29 (17)	10 (9)	55 (13)	1.92 (0.95 to 3.89)	0.07	**2**.**47 (1**.**29 to 4**.**73)**	**0**.**006**	1.10 (0.48 to 2.50)	0.83	1.78 (0.99 to 3.19)	0.05
**TNF-α G-308A**													
GG	91 (88)	52 (76)	57 (68)	52 (88)	161 (76)	1	Ref	1	Ref	1	Ref	1	Ref
GA	10 (10)	15 (22)	24 (28)	7 (12)	46 (22)	**2**.**65 (1**.**11 to 6**.**33)**	**0**.**02**	**3**.**83 (1**.**70 to 8**.**60)**	**0**.**001**	1.23 (0.44 to 3.44)	0.79	0.91 (0.39 to 8.09)	0.83
AA	2 (2)	1 (2)	3 (4)	0 (0)	4 (2)	0.88 (0.07 to 9.99)	1.00	2.42 (0.39 to 14.94)	0.37	0.35 (0.01 to 7.48)	0.53	1.14 (0.20 to 6.36)	1.00
G	192 (93)	118 (87)	138 (82)	111 (94)	367 (87)	1	Ref	1	Ref	1	Ref	1	Ref
A	14 (7)	18 (13)	30 (18)	7 (6)	55 (13)	2.09 (1.00 to 4.36)	0.05	**2**.**98 (1**.**52 to 5**.**83)**	**0**.**001**	0.86 (0.33 to 2.20)	0.81	**2**.**05 (1**.**11 to 3**.**79)**	**0**.**02**

Note: Data are no. (%) of participants unless otherwise specified. UM = uncomplicated malaria; CM = cerebral malaria; MOD = multi organ dysfunction; NCSM = non cerebral severe malaria; OR = odds ratio; 95% CI = 95% confidence interval.

Severe malaria patients were further sub-categorized in to CM, MOD and NCSM and distributions of genotypes and allele were compared with UM cases. Results are shown in Table 4. Distributions of heterozygous genotype (GA), minor allele (A) were significantly higher in MOD compared to UM for both TNF-α promoter polymorphisms (G-238A and G-308A). Prevalence of TNF-α (G-308A) heterozygous genotype (GA) was significantly higher in CM cases compared to UM (P = 0.02, OR = 2.65). Comparison of haplotype distribution revealed a significant association of A-A haplotype with predisposition to SM (P = 0.047, OR = 2.33) and MOD (P = 0.011, OR = 3.35) development (Data not shown).

### Severe malaria patients displayed higher plasma TNF-α than uncomplicated cases

**Figure 3:**
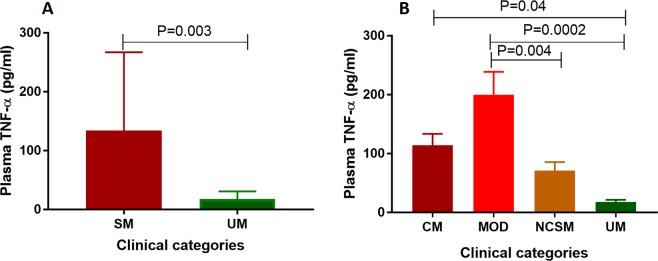
Plasma TNF-α in different clinical categories of *P*. *falciparum* malaria. (**A**) Plasma TNF-α levels was quantified by ELISA in uncomplicated malaria cases (UM) (n = 12) and severe malaria patients (SM) (n = 55) and the mean TNF-α values were compared by student’s t test. Severe malarial cases displayed significantly higher TNF-α levels compared to uncomplicated malaria (P = 0.0003). (**B**) Severe malaria cases were further categorized clinically into four sub groups viz. cerebral malaria (CM, n = 16), multi organ dysfunction (MOD, n = 21), non-cerebral severe malaria (NCSM, n = 18) and mean TNF-α levels was compared among them. P value less than 0.05 was considered as significant.

Severe malaria patients displayed significantly higher plasma TNF-α when compared to uncomplicated *P*. *falciparum* infected patients (P = 0.003) (Fig. 3A). Based on various organs involvement, severe malaria patients were further categorized into a) CM [n = 16], b) MOD [n = 21] c) NCSM [n = 18] and compared with uncomplicated cases. Patients with MOD displayed significantly higher levels of plasma TNF-α compared to NCSM (P = 0.004) and UM (P = 0.0002). In addition, a significant difference in mean plasma levels of TNF-α was also observed among CM and UM (P = 0.04) (Fig. 3B).

### *In silico* analysis

We observed a significant correlation between TNF-α polymorphisms (G-238A and G-308A) and plasma levels of TNF-α. To validate the above findings, we analyzed functional relevance of these variants *in silico*. SNPs (rs1800629 and rs361525) were submitted to the FuncPred program and results obtained are shown in Supplementary Table 2. Both the SNPs were found to affect transcription factor binding site (TFBS). However, none of them affect miRNA binding site. SNP with ID rs1800629 was found to have a regulatory potential (RegPot) of 0.0401, which was also an indication of regulatory effects on binding and expression of gene targets.

RegulomeDB database has divided both the SNPs into two distinct categories (Category 1d and Category 4 as shown in Supplementary Table 3). rs1800629 showed RegulomeDB score of 1d and rs361525 which has minimal binding evidence (Category 4). The top ranked SNP rs1800629 had annotation for eQTL + TF binding + any motif + DNase peak and thus very likely to have regulatory functions.

### Resampling analysis

As the samples size investigated in the present study was smaller, we performed a resampling analysis and data are shown in Supplementary Table 4. TNF-α (G-308A and G-238A) variants and minor alleles were more frequent in SLE cases and lupus nephritis cases suggesting an important role of TNF-α variants with predisposition to SLE and clinical manifestations.

## Discussion

TNF-α is an important cytokine in the pathogenesis as well as control of *P*. *falciparum *infection^16^. Therefore, higher levels observed in malaria infection is a protective phenomenon but very high levels can contribute to severity and mortality. The role of TNF-α in SLE is still conjectural but there are studies implicating it as a contributory factor in the pathogenesis based on experimental and associational studies^6,55^. It is important to understand the link between TNF-α and SLE in patients residing in malarial endemic areas. In the present study, we observed elevated plasma levels of TNF-α in SLE patients. Furthermore, TNF-α promoter polymorphisms (G-238A and G-308A) were significantly associated with higher plasma levels. These observations provide evidence of a possible role for TNF-α in the pathogenesis of SLE but the precise mechanism(s) is not yet known^6,55^. TNF-α is a pleotropic cytokine and acts at multiple levels^1^. In genetically susceptible SLE individuals, malaria might be a trigger for increased production of TNF-α, besides other cytokines, triggering a cascade of events contributing to the development of SLE^10,56–58^.

*P*. *falciparum* malaria is predominantly endemic in the Eastern and Northeastern parts of India^59^. But it remains endemic in most parts of the country. Prevalence of TNF-α promoter polymorphisms (G-238A and G-308A) have been reported in Indian population. Distributions of TNF-α (G-238A and G-308A) genotypes were comparable with previous reports from different parts of the country^60^,- South-West^61^, North^62^ and North-West^63^ regions. In most of the earlier reports^10,60–63^ distribution of TNF-α promoter variants were compatible with HWE in healthy controls. In the present study distribution of TNF-α (G-308A) genotypes deviated from HWE in healthy females (P = 0.0007). The geographical area of Odisha is highly endemic for *P*. *falciparum* malaria which contributes to high mortality due to malaria in the country^64^. Deviation of genotype distribution has been attributed to several factors, and selection pressure remains one of the important causes^65^. The studied population is endemic to various infectious diseases other than malaria and could be the reason for increased selection pressure on host genome^66^. Interestingly, two independent studies from South^67^ and North India^68^ have reported higher prevalence of heterozygotes (GA) compared to homozygous (GG or AA). They have also shown a deviation of TNF-α G-308A genotypes from HWE. These abnormalities in observations could be due to genotyping methods (ARMS PCR/sequence specific primer PCR) which could give spurious results.

Role of TNF-α promoter variants in SLE have been widely investigated. A recent meta-analysis including 41 published studies worldwide, showed association of minor allele (A) and AA genotype of TNF-α (G-308A) polymorphism with susceptibility to development of SLE^11^. In the present analysis, we observed higher prevalance of heterozygous (GA) and minor allele (A) in SLE patients compared to healthy females, suggesting a possible role of TNF-α (G-308A) polymorphism in susceptible to SLE. Similar observations have been reported in SLE patients from different geographical areas such as Brazil, Colombo, Mexico, North America, Spain, Taiwan^11^ and South India^10^. However, contradictory results have also been reported in Portugese, Thai, Chinese, Italian, African American, Japanese and Argentenian populations^11^. These discrepancies have been attributed to ethnicity of subjects enrolled for case-control studies and further supported by ethnicity related meta-analysis which revealed significant link between allele ‘A’ with predisposition to SLE in Europeans, Asians and South and North Americans but surprisingly not in African population^11^. In the present study, TNF-α (G-238A) heterozygous and minor allele (A) were also associated with susceptibility to SLE and it corroborated with other observations. Although the exact mechanism related to TNF-α polymorphism and SLE is yet to be understood, results of the present study and previous reports across the world indicates a strong association of TNF-α promoter variants, higher expression of TNF-α m-RNA and elevated levels of plasma TNF-α in SLE patients from malaria endemic regions.

We analysed the possible association of TNF-α polymorphisms with clinical manifestations of SLE, namely lupus nephritis which is one of the major clinical phenotypes linked to SLE mortality. We observed that heterozygous (GA) and minor allele ‘A’ of TNF-α (G-238A) polymorphism were significantly associated with lupus nephritis. These observations have been corroborated by a recent study on Chinese SLE patients^11^. However it contradicts an observation from South Indian population^10^ and a recent meta-analysis^11^. Furthermore, patients with lupus nephritis had higher levels of plasma TNF-α than those without nephritis.

Functional relevance of TNF-α promoter polymorphisms (G-308A and G-238A) have not been widely investigated. Minor allele for TNF-α (G-308A) polymorphism has been observed to enhance the binding of transcription factors and is associated with increase in mRNA production compared to major allele (G)^69^. *In vitro* stimulation of peripheral blood mononuclear cells (PBMC) derived from heterozygous subjects (GA) with lipopolysaccharide, displayed higher TNF-α than those of wild type individuals (GG)^70^. Furthermore, elevated plasma TNF-α has been associated with mutants for TNF-α (G-308A) polymorphism^10^. In the present study, we observed higher plasma levels of TNF-α in GA and AA genotypes compared to GG, corroborating earlier observations. Interestingly, other TNF-α promoter polymorphism (G-238A) also revealed similar results: mutants (GA and AA) were associated with higher plasma TNF-α than wild type (GG), which corroborates with an earlier report^71^. Furthermore, we performed *in silico* analysis which revealed regulatory effect in binding of transcription factors and enhanced expression of TNF-α gene. Results of the present investigation and earlier reports demonstrate signifcant regulatory role of promoter polymorphisms.

Investigations on possible link between malaria and SLE are limited and contradictory. Epidemiological data have shown lower prevalence of autoimmune diseases in areas where malaria incidence is high^72^. However, in an earlier observation, we have demonstrated protection against severe malaria and malarial death in complement receptor 1 variants and concluded a possible reason for higher prevalence of CR1 mutants in malaria endemic areas^14^. We had also observed that CR-1 mutants are susceptible to development of SLE and lupus nephritis since they expressed lower surface CR1 which affects clearance of apoptotic debris^73^. Furthermore, similar association of FcγRIIb variant (codon 232) with susceptibility to SLE and protection against *P*. *falciparum* malaria has been reported^74^. Lower parasitaemia and minimal clinical severity has been reported in FcγRIIb deficient mice when infected with non-lethal murine *plasmodium* strain indicating protective nature of the truncated or deficient FcγRIIb against malaria^74^. This observation has been further supported by higher prevalence of FcγRIIb codon 232 mutant in African and Asian population when compared to other populations across the world where malaria is endemic. The results of the present study and earlier reports collectively demonstrate that certain genotypes are beneficial in protecting humans against *P*. *falciparum* malaria and are highly prevalent in malaria endemic areas. Unfortunately, subjects genetically susceptible to SLE and residing in malarial endemic areas have a greater chance to develop SLE compared to those residing in non-endemic areas.

In conclusion, elevated plasma TNF-α is observed in SLE patients and associated with clinical severity. Furthermore, promoter variants of TNF-α gene, associated with higher TNF-α expression, were more prevalent in SLE patients. TNF-α is essential for clearance of malarial parasites^49^ and people residing in malarial endemic areas often produce optimal levels of TNF-α^19^ which could be helpful in combating the infection. It could also be one of the contributory factors for inducing SLE in genetically susceptible individuals. Further studies from other malarial endemic areas in the world are important to validate our findings.

## Materials and Methods

### Subjects

Gender wise analysis has been recommended in numerous earlier reports of genetic association studies^73,75,76^. SLE is a chronic inflammatory autoimmune disorder and mostly prevalent in females^77^. In the present study, we enrolled 428 female subjects (224 healthy controls and 204 SLE patients) to investigate possible association of TNF-α polymorphism in SLE. Patients of SLE were diagnosed based on the revised American College of Rheumatology (ACR) classification criteria^78^ and analyzed based on various clinical manifestations (Table 1). In addition, we enrolled 314 *P*. *falciparum* infected patients who reported to or were admitted to Department of Medicine, SCB Medical College, Cuttack, Odisha. Clinical categorization of *falciparum* infected patients were performed as described earlier^14,15,73,79^. Healthy females, age matched and residing in the same geographical areas, with no prior history of autoimmune disorders were enrolled as controls (HC). About 5 ml blood was collected from each participant. Plasma was separated and stored at −80 degrees centigrade for later use. The study was approved by the Institutional Human Ethics Committee of Central University of Jharkhand, India and S.C.B. Medical College Cuttack, Odisha, India. Informed written consent was obtained from each patient. The study was conducted in accordance with methods approved by IHECs.

### DNA isolation and genotyping of TNF-α (G-238A and G-308A) polymorphisms

Whole genomic DNA was purified from blood samples using Gen Elute Blood Genomic DNA mini Kit (Sigma-Aldrich) according to manufacturer’s instructions. TNF-α promoter polymorphisms (G-238A & G-308A) were genotyped by polymerase chain reaction followed by restriction fragment length polymorphism method as described earlier (Galbraith *et al*. 1998).

### TNF-α quantification

The plasma TNF-α levels in SLE patients, healthy controls and *P*. *falciparum* infected cases were quantified by enzyme linked immunosorbent assay (ELISA) kit (eBiosciences) according to manufacturer’s instructions.

### Non-coding SNP functional analysis

In order to recognize the effect of SNPs in non-coding regions, tools predicting probable functional effect of SNPs at transcription factor binding sites (TFBS), Intron/exon border consensus sequences (splice sites), Exonic splicing enhancers (ESEs), and miRNA binding were utilized. SNPinfo (FuncPred) and RegulomeDB offer a pool of functional information using series of tools. The SNPs functionality was defined by SNPinfo (FuncPred) (https://snpinfo.niehs.nih.gov/snpinfo/snpfunc.php) web server^80^, which helps in selecting SNPs for genetic association studies. For the current study, two SNPs (rs1800629 and rs361525) were uploaded for batch analysis with the default settings. The output information was a list of SNPs with possible functional effect.

To supplement SNP ranking, SNPs were further analyzed by RegulomeDB (http://regulomedb.org/)^81^. RegulomeDB categorizes variants into six categories ranging from 1 to 6, where category 1 variants are ‘likely to affect binding and linked to expression of a gene target’, category 2 variants are ‘likely to affect binding’, Category 3 variants are ‘less likely to affect binding’, and Category 4, 5 and 6 variants have ‘minimal binding evidence’. RegulomeDB also allocates a score of 7 for variants with no annotation data available. dbSNP rsIDs were utilized as input for the current study.

### Statistical analysis

Genotype and allele distribution among different clinical categories was compared by Fisher’s exact test. P value less than 0.02 was taken as significant (Bonferroni correction for two SNPs 0.05/2 = 0.02). The mean plasma levels of TNF-α in SLE patients and healthy controls was compared by student’s t test and analysis of variance (ANOVA) was employed for study difference in plasma TNF-α in different clinical categories of *P*. *falciparum* malaria. The association of TNF-α (G-238A and G-308A) genotypes with plasma TNF-α levels were analyzed by unpaired ‘t’ test or ANOVA followed by an appropriate post-test. Graph Pad Prism 5.01 software was used for these statistical analyses. Haplotype analysis was performed by SNAP Stats online tool. Resampling analysis was performed by bootstrap method in Microsoft excel sheet attached as supplementary file-1.

## Supplementary information

Supplementary file

Supplementary Dataset-1

## Data Availability

The datasets generated during and/or analysed during the current study are available from the corresponding author on reasonable request.
