# Supplementary material for: TNF-α promoter polymorphisms (G-238A and G-308A) are associated with susceptibility to Systemic Lupus Erythematosus (SLE) and P. falciparum malaria: a study in malaria endemic area
Source: Sci Rep. 2019 Aug 13;9:11752. doi: 10.1038/s41598-019-48182-5 (PMC6692415; doi:10.1038/s41598-019-48182-5)
Supplement: Supplementary file 1 — Supplementary file [file 41598_2019_48182_MOESM1_ESM.pdf]

Supplementary Informations

Title: TNF-α promoter polymorphisms (G-238A and G-308A) are associated with susceptibility to Systemic Lupus Erythematosus (SLE) and *P. falciparum* malaria: a study in malaria endemic area.

Harishankar Mahto[1], Rina Tripathy[2], Biswa Ranjan Meher[3#], Birendra K Prusty[4], Meenakshi Sharma[1], Divya Deogharia[1], Anjana Kumari Saha[1], Aditya K Panda[1*#], Bidyut K Das[5*]

Supplementary Table 1: Haplotype analysis of TNF-α polymorphisms (G-238A and G-308A) in SLE patients and Healthy control

Haplotype (G-238A/G-308A)	HC n=204	SLE n=224	P value	OR (95% CI)
G-G	86.96	78.84	--	1
A-G	05.22	06.70	0.25	1.41 (0.78-2.55)
G-A	05.89	09.64	0.049	1.63 (1.00-2.66)
A-A	01.92	04.82	0.029	2.57 (1.11-5.96)

Note: Prevalance of halplotype data are in percentage. HC = healthy control; SLE = systemic lupus erythematosus; OR = odds ratio; 95% CI = 95% confidence interval.

Supplementary Table-2. SNPs predicted by FuncPred (SNPinfo) and having functional effect.

SNP	dbSNP ID	Chromosome	position	Allele	RegPotential	TFBS	miRNA	Distance (bp)
G-238A	rs361525	6	31651080	A/G	0.0	Y	-	-1003||-249
G-308A	rs1800629	6	31651010	A/G	0.040179	Y	-	-933||-319

TFBS: transcription factor binding site,

RegPotential: Regulatory Potential, that measures the similarity of patterns in the alignments to those in known regulatory regions.

Y: SNPs which affect function,

– : SNPs which does not affect function

Supplementary Table-3. SNPs category and description based on RegulomeDB score.

SNP	dbSNP ID	RegulomeDB Score	Category	Description
G-238A	rs361525	4	Minimal binding evidence	TF binding + DNase peak
G-308A	rs1800629	1d	Likely to affect binding and linked to expression of a gene target	eQTL + TF binding + any motif + DNase peak

TF: transcription factor,

eQTL: expression quantitative trait loci

Score	
1a	eQTL + TF binding + matched TF motif + matched DNase Footprint + DNase peak
1b	eQTL + TF binding + any motif + DNase Footprint + DNase peak
1c	eQTL + TF binding + matched TF motif + DNase peak
1d	eQTL + TF binding + any motif + DNase peak
1e	eQTL + TF binding + matched TF motif
1f	eQTL + TF binding / DNase peak
2a	TF binding + matched TF motif + matched DNase Footprint + DNase peak
2b	TF binding + any motif + DNase Footprint + DNase peak
2c	TF binding + matched TF motif + DNase peak
3a	TF binding + any motif + DNase peak
3b	TF binding + matched TF motif
4	TF binding + DNase peak
5	TF binding or DNase peak
6	Other

Source: http://www.regulomedb.org

Supplementary Table-4 Analysis of 1000 re-sampling groups for association of TNF-a polymorphisms (G-308A and G-238A) with SLE and lupus nephritis.

Genotype/allele	HC	SLE	OR	95% CI	P value
G-308A					
GG	194236	152718	ref	--	1
GA	25032	42914	2.18	2.14 to 2.21	<0.0001
AA	5034	8066	2.03	1.96 to 2.11	<0.0001
G	413504	348350	ref	--	1
A	35100	59046	1.99	1.96 to 2.02	<0.0001
G-238A					
GG	195096	159216	ref	--	1
GA	25719	43042	2.05	2.01 to 2.08	<0.0001
AA	2907	2020	0.85	0.80 to 0.90	<0.0001
G	415911	361474	ref	--	1
A	31533	47082	1.71	1.69 to 1.74	<0.0001
	LN-	LN+			
G-308A					
GG	96733	56260	ref	--	1
GA	18982	23777	2.15	2.10 to 2.20	<0.0001
AA	5161	3087	1.02	0.98 to 1.07	0.23
G	212448	136297	ref	--	1
A	29304	29951	1.59	1.56 to 1.62	<0.0001
G-238A					
GG	103957	55003	ref	--	1
GA	17140	25934	2.86	2.79 to 2.92	<0.0001
AA	0	1966			
G	225054	135940	ref	--	1
A	17140	29866	2.88	2.82 to 2.94	<0.0001

Note: Data are number of subjects calculated by 1000 re-sampling groups. HC= healthy controls; SLE: systemic lupus erythematosus; LN^+ = lupus nephritis; LN^- = non-lupus nephritis; OR = odds ratio; 95% CI = 95% confidence interval.
